# Sensitivity to Information Conveyed by Horizontal Contours is Correlated with Face Identification Accuracy

**DOI:** 10.3389/fpsyg.2013.00074

**Published:** 2013-02-25

**Authors:** Matthew V. Pachai, Allison B. Sekuler, Patrick J. Bennett

**Affiliations:** ^1^Department of Psychology, Neuroscience, and Behaviour, McMaster UniversityHamilton, ON, Canada; ^2^Centre for Vision Research, York UniversityToronto, ON, Canada

**Keywords:** face perception, face identification, masking, orientation tuning, ideal observer, face inversion effect

## Abstract

We measured thresholds in a 1-of-10 face identification task in which stimuli were embedded in orientation-filtered Gaussian noise. For upright faces, the threshold elevation produced by the masking noise varied as a function of noise orientation: significantly greater masking was obtained with horizontal noise than with vertical noise. However, the orientation selectivity of masking was significantly less with inverted faces. The performance of an ideal observer was qualitatively similar to human observers viewing upright faces: the masking function exhibited a peak for horizontally oriented noise although the selectivity of masking was greater than what was observed in human observers. These results imply that significantly more information about facial identity was conveyed by horizontal contours than by vertical contours, and that human observers use this information more efficiently to identify upright faces than inverted faces. We also found a significant positive correlation between selectivity for horizontal information and face identification accuracy for upright, but not inverted faces. Finally, there was a significant positive correlation between horizontal tuning and the size of the face inversion effect. These results demonstrate that the use of information conveyed by horizontal contours is associated with face identification accuracy and the magnitude of the face inversion effect.

## 1 Introduction

We detect, discriminate, and recognize hundreds of faces every day. However, despite the apparent ease with which face recognition normally operates, there are some conditions in which we experience difficulty. For example, rotating a face 180° in the picture plane significantly impairs recognition, and these effects of rotation appear to be larger for faces than for other kinds of objects (Yin, 1969; Valentine, 1988; Husk et al., 2007). This well-established face inversion effect is interesting because the physical information available to discriminate two inverted faces is the same as that available to discriminate two upright faces, and therefore a difference in perceptual processing or observer strategies must underlie the face inversion effect.

The cause of the inversion effect remains a matter of debate. One of the most commonly held theories is that upright and inverted faces are processed using qualitatively different mechanisms: with holistic/configural mechanisms dominating for upright, but not inverted faces (Diamond and Carey, 1986; Young et al., 1987; Tanaka and Farah, 1993; Farah et al., 1995; Rossion, 2008). However, it has also been suggested that upright and inverted face processing differs quantitatively, not qualitatively: in effect, that upright faces are processed more efficiently than inverted faces (Valentine, 1988; Riesenhuber et al., 2004; Sekuler et al., 2004; Yovel and Kanwisher, 2004). Using the classification image technique, Sekuler et al. (2004) found that observers relied on information carried by pixels near the eyes and eyebrows to identify both upright and inverted faces. Based on this result, Sekuler et al. suggested that observers use similar spatial regions to identify upright and inverted faces, but that inversion produces a quantitative difference in the ability to extract relevant information from those regions. Gaspar et al. (2008a) tested this hypothesis directly using the equivalent noise paradigm, and found that inversion decreased calculation efficiency alone, supporting the notion that observers simply use available physical information in the stimuli less effectively when processing inverted faces.

What then leads to decreased processing efficiency for inverted faces compared to upright faces? One possibility is that different spatial frequencies are used to identify upright and inverted faces; however, direct comparisons of the spatial frequency tuning of upright and inverted face identification reveal that observers rely on similar spatial frequencies in both cases (Gaspar et al., 2008b; Willenbockel et al., 2010). It is unlikely, therefore, that the face inversion effect is caused by observers using different bands of spatial frequencies to identify upright and inverted faces.

**Figure 1:**
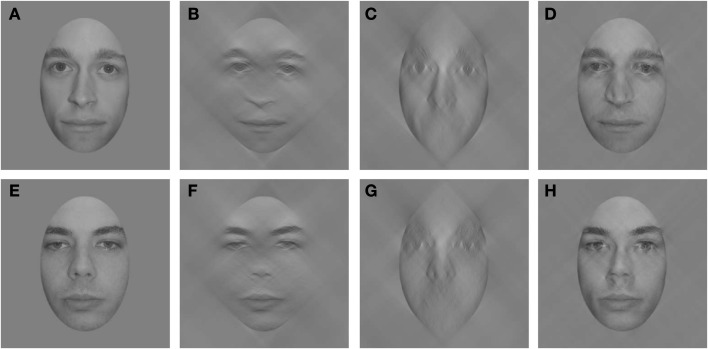
**Two faces (A,E) filtered to retain only horizontal (B,F) or vertical (C,G) information (bandwidth = 90°)**. Hybrid faces **(D,H)** constructed using horizontal information from one face and vertical information from the other resemble the face from which the horizontal information is drawn (D = B + G and H = F + C). Note that equating the RMS contrast of the filtered components has a negligible effect on the hybrids.

Spatial frequency selectivity is, of course, only one way that identification may differ for upright and inverted faces. Recently, Dakin and Watt (2009) demonstrated that orientation information, specifically conveyed by horizontal contours, may be especially useful for face identification (see also Figure 1). Given this finding, perhaps a difference in the use of horizontal information may explain the performance deficits incurred following face inversion. Goffaux and Dakin (2010) examined this hypothesis with face stimuli filtered to contain narrow bands of orientations centered on horizontal, vertical, or both orientations. Using a same/different paradigm, Goffaux and Dakin found that performance was better for upright faces containing horizontal information than upright faces containing vertical information. However, when the faces were inverted, overall performance decreased and the horizontal advantage disappeared. This result demonstrates that discrimination of upright faces is indeed supported by the use of horizontal information, but this orientation difference disappears when the face is inverted. In a series of additional experiments, Goffaux and Dakin (2010) demonstrated the importance of horizontal information for other face phenomena such as identity aftereffects, viewpoint-invariance, and holistic processing. However, it remains unclear whether the importance of horizontal contours reflects the additional diagnostic information conveyed by that orientation (Dakin and Watt, 2009) or by observers processing that orientation more efficiently.

The primary goal of this study was to disentangle the preferential use of horizontal information by human observers from the informational structure of the stimulus. To this end, we employed a 10-AFC face identification task in which the stimuli were masked with orientation-filtered Gaussian noise. This approach allowed us to assess the importance of different orientation bands by measuring the decrement in identification performance incurred when they are masked. We also used an ideal observer analysis to systematically assess the information available at each orientation band. An ideal observer is an optimal decision maker that achieves the best possible performance on a task given the available stimulus information (Bennett and Banks, 1987; Banks et al., 1991; Tjan et al., 1995). By measuring the effect of orientation-filtered noise on ideal performance, we obtained measures of how much diagnostic information is carried by different bands of orientations, which in turn allowed us to estimate how efficiently human observers used the available information at different orientations.

Based on the findings of Dakin and Watt (2009), we predicted that the ideal observer would show larger masking effects for horizontally oriented noise with a decrease in masking at off-horizontal orientations. Moreover, based on the findings of Goffaux and Dakin (2010) we expected human observers to show horizontally peaked masking, similar to the ideal observer, for upright but not inverted face stimuli. We also examined whether individual differences in face identification accuracy and/or the face inversion effect (Bruce et al., 1999; Sekuler et al., 2004; Konar et al., 2010) can be explained by differences in the use of horizontal information. Specifically, if the preferential use of horizontal information is associated with face identification, then there ought to be a positive correlation between face identification accuracy and the strength of horizontal tuning.

## 2 Materials and Methods

### 2.1 Observers

Thirty-two observers (9 male, 23 female; average age 21 years) participated in the experiment. All observers were naïve to the purpose of the experiment and had normal or corrected-to-normal Snellen acuity. Observers were paid $10/h or given course credit for their participation. All experimental protocols were approved by the McMaster University Research Ethics Board, and informed consent was collected prior to initiation of the experiment.

### 2.2 Stimuli

Stimuli were generated on an Apple Macintosh G4 computer using MATLAB and the Psychophysics and Video Toolboxes (Brainard, 1997; Pelli, 1997). Stimuli were presented on a 21″ Apple Studio display with a resolution of 1280 × 1024 pixels and a frame rate of 85 Hz. Average luminance, which was 30.8 cd/m^2^, was held constant throughout the experiment. The face stimuli were based on digitized photographs of 5 male and 5 female models (average age 24 years) with no visible piercings, facial hair, or eye glasses. Models were photographed as they turned their head to face a variety of gaze directions, each separated by 4.5° of visual angle. In this way, each identity was represented by a variety of images with viewpoints to the left and the right, as well as one frontal view. Each photograph was cropped to remove external features such as hair, ears, and chin. The faces were centered in a 372 × 372 pixel matrix which subtended 4.6° × 4.6° at the viewing distance of 60 cm. See Gaspar et al. (2008b) for more details about the stimuli.

**Figure 2:**
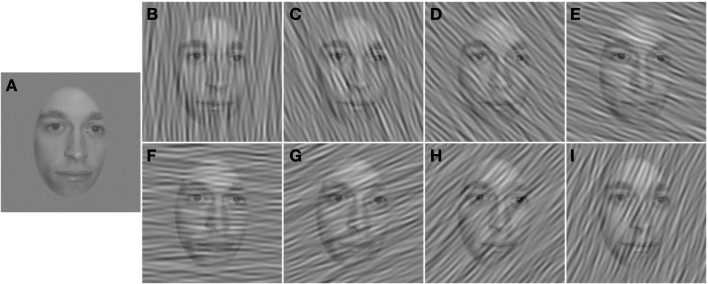
**High-contrast examples of stimuli in each condition**. **(A)** White noise only. **(B–I)** White noise and orientation-filtered noise with center orientations ranging from −90° **(B)** to 67.5° **(I)** in 22.5° steps.

Two independent Gaussian noise fields were added to the stimulus on every trial. One was an unfiltered (i.e., white) Gaussian noise with an RMS contrast of 0.028. The other noise field was filtered with an ideal, band-pass orientation filter with a full bandwidth of 23° centered at one of eight orientations ranging from −90° (vertical), through 0° (horizontal), to 67.5° in 22.5° steps. The RMS contrast of the filtered noise was 0.14 prior to filtering. In a ninth condition, the contrast of the filtered noise was set to zero, so that the stimuli were embedded only in unfiltered, white Gaussian noise. Figure 2 demonstrates a stimulus masked in each of the different orientation conditions.

### 2.3 Procedure

Participants viewed the display binocularly, and a chin/head rest was used to stabilize the viewing position. Each trial began with a small, high-contrast fixation point presented at the center of the screen for 500 ms. The fixation point was extinguished and, after a delay of 200 ms, a face embedded in noise was presented for 250 ms. On each trial, a random viewpoint was selected for the current identity to discourage the use of simple image-matching strategies. Following the stimulus, a response selection screen containing noise-free, high-contrast (RMS contrast = 0.3) frontal views of the 10 face identities was presented and the observer selected the target with a mouse click. Note that frontal views only appeared on the response selection screen; target stimuli always were presented with viewpoints to the left or the right. Feedback was provided in the form of 600 and 200 Hz tones following correct and incorrect responses, respectively.

### 2.4 Design

Observers completed the experiment over the course of two sessions, separated by approximately 24 h. Within each session, observers completed two blocks of trials: one block used upright stimuli, and the other used inverted stimuli. The order of face orientation blocks was counter-balanced across observers. The orientation of the faces in the response selection screen was the same as the orientation of the target stimuli. Noise conditions (eight orientation-filtered noises plus one white noise) were intermixed randomly within blocks. Face RMS contrast was varied across trials with the FAST toolbox, a Bayesian adaptive threshold estimator (Vul and MacLeod, 2007).

Two thresholds per noise condition were measured simultaneously within each block. A block ended when the threshold estimates for each condition were based on at least 20 trials and had a 95% confidence interval of less than 0.3 log units. Prior to the experiment, we were uncertain whether the strength of masking produced by oriented masking noise depended on the response accuracy used to define threshold. Therefore, we measured thresholds using two criterion levels of response accuracy: for 16 subjects, threshold was defined as the RMS contrast needed to achieve 67% correct responses, and for the remaining 16 subjects threshold was defined as the RMS contrast needed to achieve 50% correct. In the following sections we refer to these two groups as the *t*_67_ and *t*_50_ groups.

### 2.5 Data analysis

The two thresholds for each condition in each session were averaged to form a single dependent measure. In each block, orientation masking was defined as the threshold obtained with an orientation-filtered noise divided by the threshold in the unfiltered (i.e., white) noise condition. These masking ratios were log-transformed prior to analysis.

### 2.6 Ideal observer analysis

The ideal observer is an optimal decision maker that achieves the best possible performance on a task given the available stimulus information (Bennett and Banks, 1987; Banks et al., 1991; Tjan et al., 1995). If information within a given orientation band is not relevant for the task, then the performance of the ideal observer should not vary when that orientation band is masked. On the other hand, if information in a given orientation band is critical for the task, then performance of the ideal observer should be impaired when that orientation band is masked.

For a task like ours that uses white noise, the ideal observer is a cross-correlator that measures the *a posteriori* probability of each stimulus identity given a particular noisy input (Tjan et al., 1995). If *R* is the noisy stimulus, σ^2^ is the variance of the noise, *T_ij_* is the *j*th view of the *i*th identity, and *P*(*T_ij_*) is the *a priori* probability of being shown *T_ij_*, then the ideal observer selects the identity *i* that maximizes the function.

(1) $$\sum_{j}\text{exp}\left(-\frac{1}{2\sigma^{2}}{∥R-T_{ij}∥}^{2}\right)P\left(T_{ij}\right)$$

where ||*R* − *T_ij_*||^2^ is defined as the Euclidian distance between the image and the template, and is equivalent to maximizing the cross correlation *RT_ij_* between the stimulus and template when all the templates contain the same energy (Tjan et al., 1995).

Our experiments used filtered noise, and therefore the ideal observer used templates that were adjusted to take into account the fact that noise power varies as a function of orientation. This adjustment can be carried out by computing the product, in the Fourier domain, of the original template, and a pre-whitening filter that removes the noise correlations in the stimulus (Myers et al., 1985; Eckstein et al., 1997). These adjusted templates were used to maximize equation (1).

We used computer simulations to calculate the performance of the ideal observer on our task. The stimuli, procedure, and design were identical to those used in the main experiment with the exception that we did not include an inverted condition because the ideal observer’s performance is identical for upright and inverted faces. We simulated 10 sessions, yielding a total of 20 thresholds per condition. The mean of the 20 thresholds in each condition was calculated and utilized for all subsequent analyses.

## 3 Results

All statistical analyses were performed with R (R Development Core Team, 2012). The Huynh-Feldt correction, $\tilde{\varepsilon },$ was used to adjust *p* values of *F* tests conducted with within-subject variables to correct for violations of sphericity (Maxwell and Delaney, 2004).

### 3.1 Ideal observer

**Figure 3:**
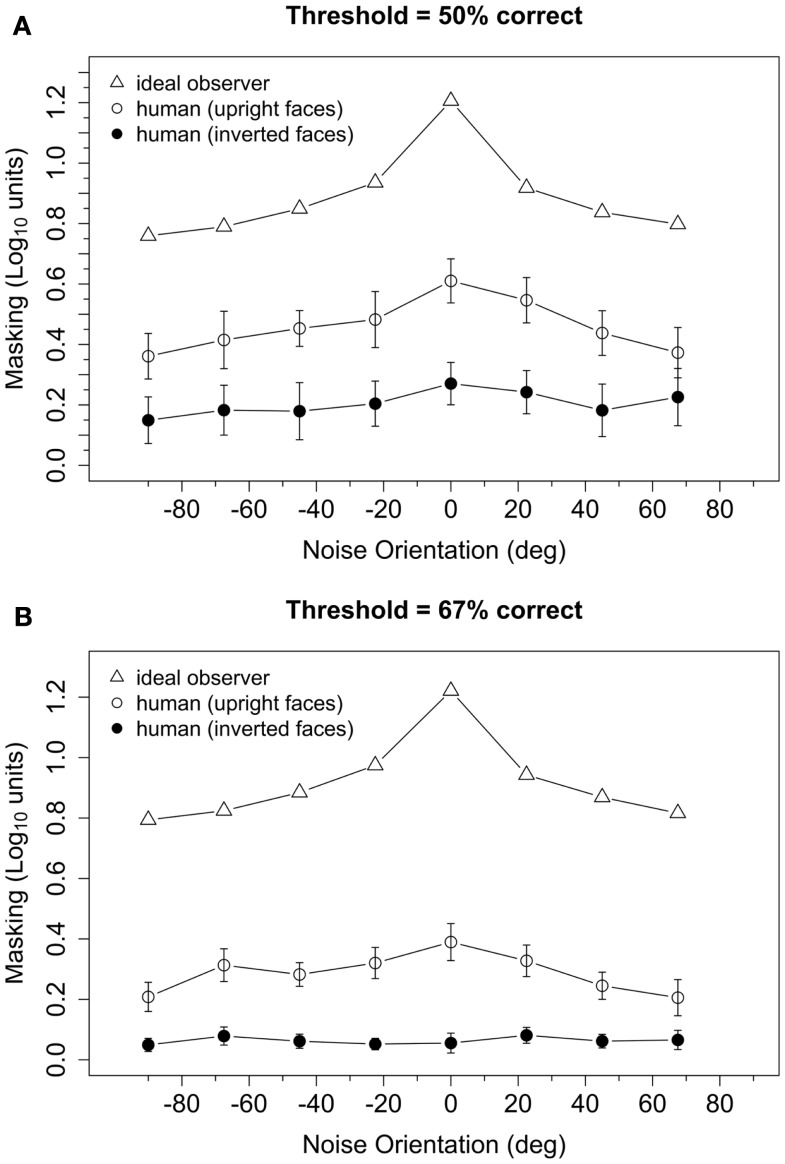
**Log-transformed masking ratios plotted as a function of noise orientation for the ideal observer and human observers with upright and inverted face stimuli**. Threshold was defined as the RMS contrast needed to achieve 50% **(A)** or 67% **(B)** correct responses, and the masking ratio was defined as the mean of the log-transformed ratios of masked to unmasked contrast thresholds. Error bars represent ±1 SEM.

Figure 3 plots log-transformed masking ratios as a function of noise orientation for the ideal and human observers. Consider first the ideal observer. When a particular orientation is masked by filtered noise, the ideal observer is forced to rely more heavily on information carried in the other orientation bands. Hence, the amount of masking obtained for each noise orientation is related to the amount of face identification information carried in each orientation band. Obtaining no masking would indicate that no information is carried in that orientation band, whereas a large masking ratio indicates that significant information is carried in that band. The ideal masking ratios in Figure 3 indicate that the amount of identification information was greatest for orientations near 0° (i.e., horizontal), least for orientations near ±90° (vertical), and intermediate for orientations near ±22.5°, ±45°, and ±67°. This result highlights the fact that different orientations do, in fact, carry different amounts of physical information for our face discrimination task, as previously suggested by Dakin and Watt (2009). Specifically, there is more information for identification in the physical stimulus around horizontal orientations than around vertical orientations.

### 3.2 Human observers

Masking obtained from human observers as a function of noise orientation with upright and inverted faces is shown in Figure 3. A preliminary analysis indicated that log-transformed masking ratios, averaged across noise orientations, were greater than zero at both threshold criteria and both face orientations (Table 1). However, inspection of Figure 3 suggests that masking was greater in the *t*_50_ group than in the *t*_67_ group, and greater for upright faces than inverted faces. Furthermore, masking obtained with upright faces appeared to vary systematically with noise orientation, but was nearly independent of noise orientation with inverted faces. These observations were confirmed by a 2 (threshold criteria) × 2 (face orientation) × 8 (noise orientation) ANOVA performed on log-transformed masking ratios: the main effects of threshold criteria [*F*(1,30) = 6.38, *p* = 0.017], face orientation [F(1,30) = 20.54, *p* < 0.001], and noise orientation [F(7,210) = 8.28, *p* < 0.001] were significant, as was the interaction between face and noise orientation [F(7,210) = 4.51, *p* = 0.001].

**Table 1:** **Mean and 95% confidence intervals for masking averaged across noise orientations**.

Group	Face orientation	*M*	*CI*_95_
*t*_50_	Upright	0.418	[0.307, 0.613]
*t*_50_	Inverted	0.204	[0.045, 0.364]
*t*_67_	Upright	0.286	[0.187, 0.385]
*t*_67_	Inverted	0.063	[0.017, 0.109]

**Figure 4:**
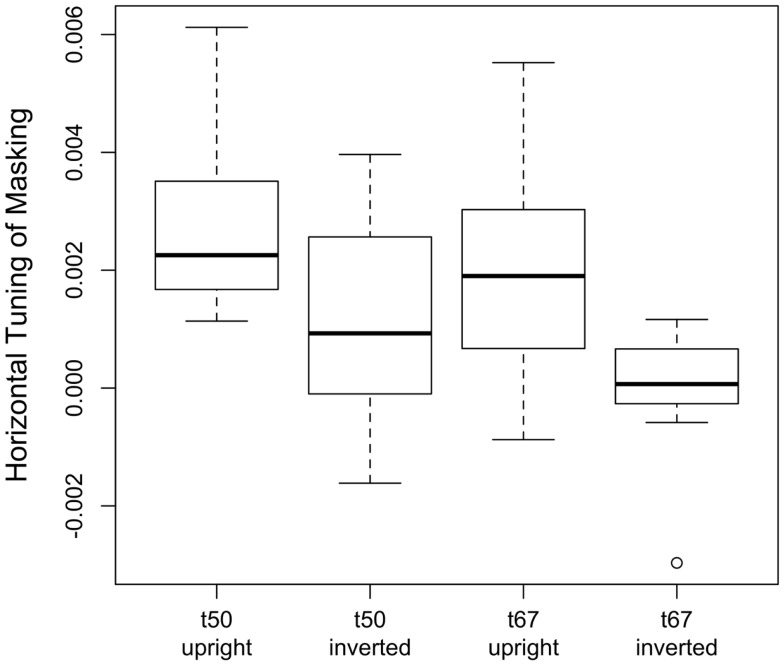
**Orientation tuning of masking for upright and inverted faces in the *t*_50_ and *t*_67_ groups**. Tuning was defined as the slope of the regression line fit to masking obtained with noise orientations of −90°, 67.5°, 45°, 22.5°, and 0°. Masking values at 67.5, 45, and 22.5 were defined as the average level of masking obtained at, respectively, ±67.5°, ±45°, and ±22.5°. The horizontal line in each boxplot indicates the median; the upper and lower edges of each box indicate the 75th and 25th percentile, respectively.

The significant interaction between face and noise orientation reflects the fact the masking functions obtained with upright faces, but not inverted faces, exhibited a peak near 0°. To quantify this interaction, we computed a measure of orientation tuning for each subject by estimating the slope of a regression line that related masking to the orientation of the noise. Initially, we fit two regression lines to the masking data: one to the ascending part of the masking function for noise orientations from −90° to 0°, and another to the descending part of the function (i.e., noise orientations from 0° to 90°). However, the slopes of the ascending and descending parts of the curve were significantly correlated [upright: *r* = 0.81, *t*(30) = 7.58, *p* < 0.001; inverted: *r* = 0.69, *t*(30) = 5.26, *p* < 0.001]. Furthermore, a 2 (face orientation) × 2 (threshold criterion) × 2 (masking function part: ascending vs. descending) ANOVA on the slopes found that the main effect of masking function part [*F*(1,30) = 2.82, *p* = 0.10], as well as all of the interactions with that factor [*F* ≤ 2.93 and *p* ≥ 0.10, in each case], were not significant. Therefore, to simplify our analyses, we averaged the two measures of masking obtained with ±22.5°, ±45°, and ±67.5° noise, computed a regression line for masking at noise orientations of −90°, ±67.5°, ±45°, ±22.5°, and 0°, and used the slope of the regression line as the index of the horizontal tuning of masking. Boxplots of slopes of the regression lines are shown in Figure 4: tuning appeared to be significantly higher for upright faces than inverted faces, and slightly higher in the *t*_50_ group than the *t*_67_ group. A 2 (face orientation) × 2 (threshold criterion) ANOVA on the horizontal tuning measures confirmed these observations: the main effects of face orientation [*F*(1,30) = 49.12, *p* < 0.001] and threshold criterion [*F*(1,30) = 4.84, *p* = 0.035] were significant, but the face orientation × threshold criterion interaction was not significant [*F*(1,30) = 0.33, *p* = 0.57]. One subject in the *t*_67_ group had an unusually low slope in the inverted face condition (Figure 4). When this subject was removed from the analysis, the main effect of face orientation was significant [*F*(1,29) = 45.42, *p* < 0.001] but the main effect of threshold criterion [*F*(1,29) = 3.60, *p* = 0.07] and interaction between face orientation and threshold criterion [*F*(1,29) = 0.27, *p* = 0.60] were not. Note that nearly identical results were obtained from separate ANOVAs that were conducted on tuning measures tuning derived from the ascending and descending parts of the masking function. Separate *t* tests indicated that tuning differed significantly from zero in all conditions [*t*(15) ≥ 2.88, *p* ≤ 0.011, in each case] except for inverted faces in the *t*_67_ group with [*t*(15) = 0.12, *p* = 0.91] or without [*t*(14) = 1.68, *p* = 0.12] the outlier. Together, these results suggest that the orientation selectivity of masking was greater for upright faces than inverted faces.

### 3.3 Absolute efficiency

Our results demonstrate that human observers used information across a wide range of orientations for upright face discrimination, with more weight being given to orientations closer to horizontal. However, as our ideal observer analysis showed, more information is available in the horizontal orientations for face discrimination. To what extent does the human pattern of results simply reflect the variation of information across orientations? We addressed this question by calculating absolute efficiency of human observers as a function of orientation for upright and inverted faces. In an identification task such as ours, absolute efficiency is defined as the squared ratio of the ideal to human RMS contrast thresholds.

**Figure 5:**
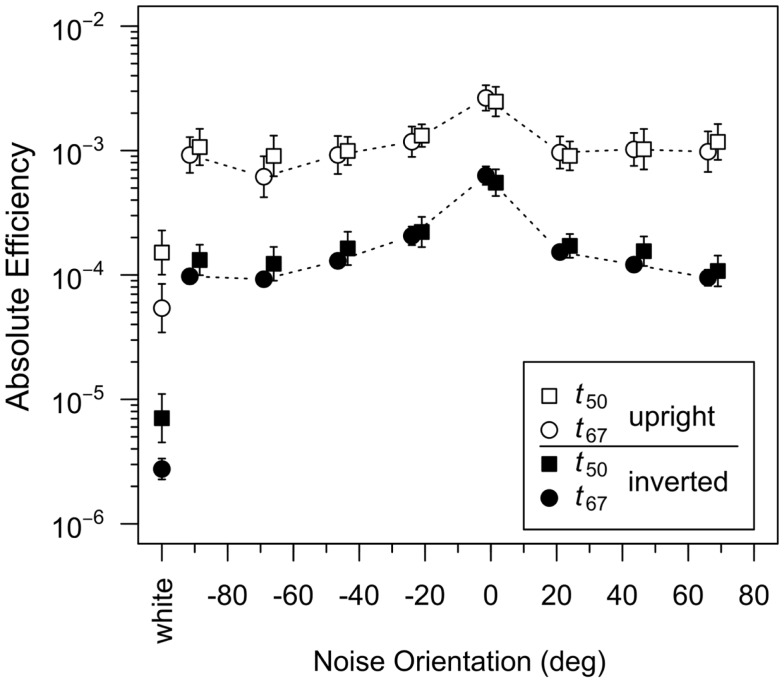
**Absolute efficiency measured in the *t*_50_ and *t*_67_ groups plotted as a function of noise orientation for upright and inverted faces**. The leftmost symbols represent efficiency in the white noise condition. Absolute efficiency is defined as the squared ratio of ideal and human RMS contrast thresholds. Points for the *t*_50_ and *t*_67_ groups have been offset slightly for clarity. Error bars, where visible, represent ±1 SEM.

If human observers extracted information from all bands of orientation with equal efficiency, then absolute efficiency for faces embedded in filtered noise ought to be constant as a function of noise orientation. On the other hand, if human observers use information at a particular orientation relatively poorly, then masking noise at that orientation should increase threshold more in the ideal observer than in human observers and therefore result in *higher* efficiency. Figure 5 plots absolute efficiency as a function of noise orientation for upright and inverted faces. Consistent with previous reports, efficiency obtained with a white noise mask was higher for upright than inverted faces (Gaspar et al., 2008a), although efficiency was quite low at both face orientations (Gold et al., 1999, 2004). A 2 (threshold criterion) × 2 (face orientation) ANOVA on log-transformed efficiency in the white noise conditions yielded significant main effects of threshold criterion [*F*(1,30) = 4.38, *p* = 0.045] and face orientation [*F*(1,30) = 110.6, *p* < 0.001]; the interaction between threshold criterion and noise orientation was not significant [*F*(1,30) = 0.024, *p* = 0.87].

With both upright and inverted faces, average efficiency was higher in conditions that used orientation-filtered noise than in the white noise condition, which is due to the fact that the addition of filtered noise increased thresholds less (i.e., masking was lower) in human observers than the ideal observer (Figure 3). As discussed previously, this result suggests that human observers used information at all orientations, albeit less efficiently than the ideal observer. Furthermore, the fact that the addition of filtered noise increased efficiency more for inverted faces than upright faces is consistent with the observation that efficiency is lower overall for inverted faces. Efficiency also varied with noise orientation, although the variation appeared greater with inverted faces. Finally, adding filtered noise eliminated the difference between efficiency in the *t*_50_ and *t*_67_ groups. These observations were confirmed with a 2 (threshold criterion) × 2 (face orientation) × 8 (noise orientation) ANOVA on log-transformed efficiency: the main effect of threshold criterion was not significant [*F*(1,30) = 0.21, *p* = 0.65], but the main effects of face orientation [*F*(1,30) = 95.13, *p* < 0.001] and noise orientation [*F*(7,210) = 41.57, $\tilde{\epsilon }=0.748,$ *p* < 0.001] were significant, as was the interaction between face and noise orientation [*F*(7,210) = 4.51, $\tilde{\epsilon }=0.939,$ *p* < 0.001]. The significant interaction reflected the fact that the difference between efficiency for upright and inverted faces was ≈1 log unit when the noise orientation was −90° and ±67.5° but only ≈0.6 log units with 0° noise. Again, because suboptimal use of a particular orientation band should result in *higher* efficiency, this interaction suggests that human observers were suboptimal in their use of horizontal information, particularly so with inverted faces. Follow up analyses indicated that the effect of noise orientation was significant for both upright [*F*(7,210) = 13.38, $\tilde{\epsilon }=0.826,$ *p* < 0.001] and inverted faces [*F*(7,210) = 39.14, $\tilde{\epsilon }=0.793,$ *p* < 0.001], suggesting that observers differ quantitatively in their use of orientation information following inversion, relying on horizontal contours in both cases, but less effectively with inverted faces.

Absolute efficiency was lower in the *t*_67_ group than the *t*_50_ group in the white noise condition but not the oriented noise conditions. The group difference in the white noise condition is consistent with the hypothesis that the psychometric function relating face contrast to response accuracy in human observers was shallower than the psychometric function for the ideal observer: contrast had to be increased more in human observers to increase accuracy from 50 to 67% correct. Conversely, the lack of a group difference in the oriented noise conditions implies that the psychometric functions for human and ideal observers had similar slopes in those conditions. However, it is unclear why the slope of the psychometric function differed in the white and oriented noise conditions.

### 3.4 Correlation analysis

**Figure 6:**
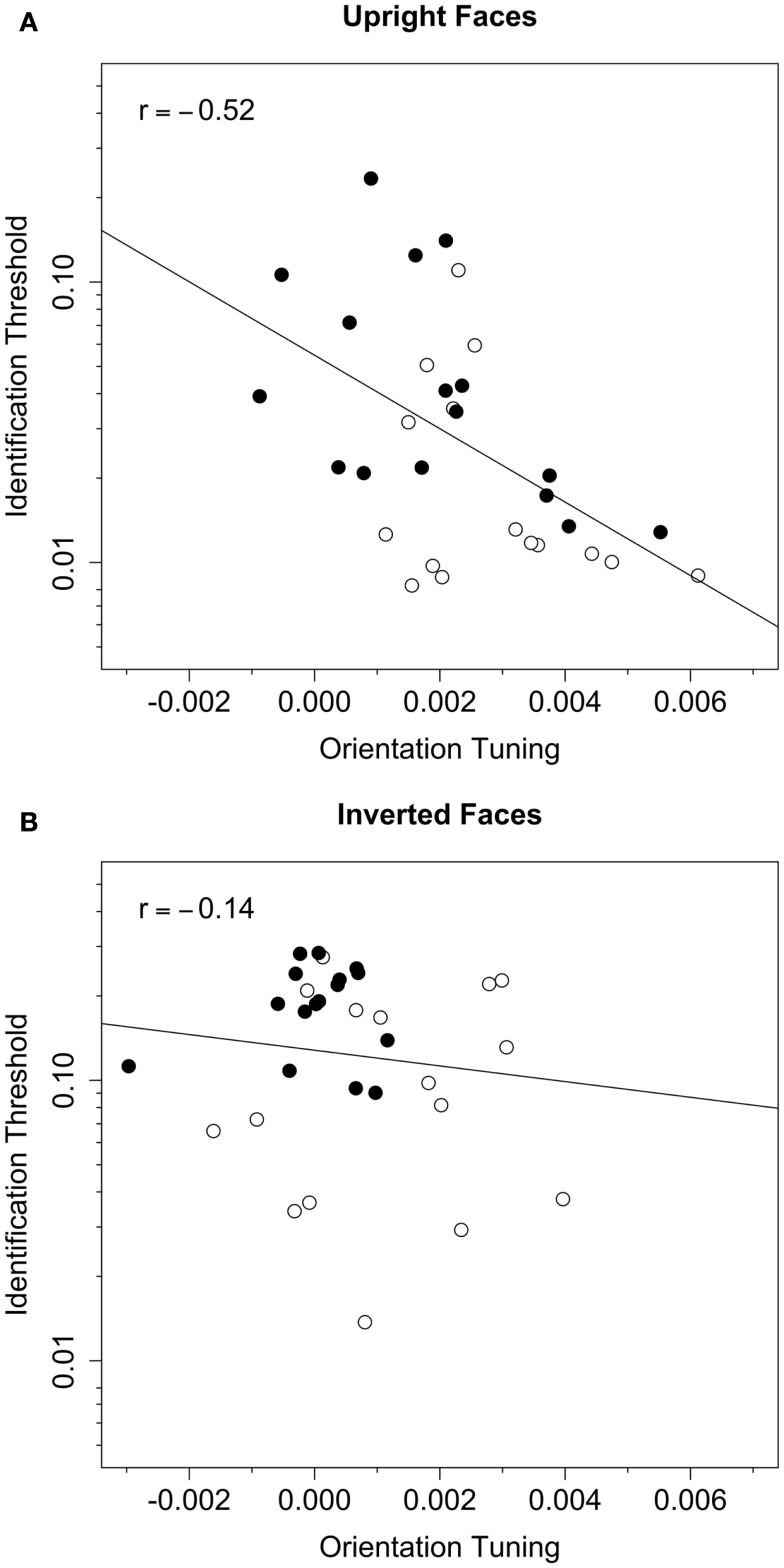
**Identification threshold plotted against orientation tuning for upright (A) and inverted (B) faces**. Data from the *t*_67_ and *t*_50_ groups are represented by the filled and open symbols, respectively. The dotted line represents the best-fitting (least-squares) line fit to the data from both groups. The Pearson correlation between identification threshold and orientation tuning was significant for upright (*r* = −0.52) but not inverted (*r* = −0.14) faces.

To assess the association between orientation tuning and face identification threshold, we evaluated linear models that included log-transformed threshold in the white noise condition as the dependent variable, and threshold criterion (i.e., *t*_67_ vs. *t*_50_), orientation tuning (see Figure 4), and the interaction between criterion and tuning as predictor variables. These models allowed us to quantify the variance in identification thresholds that could be explained by variance in each of the predictor variables. Note that the derivation of orientation tuning did not include thresholds in the white noise condition, and therefore the two variables were not necessarily related. Models for upright and inverted faces were evaluated separately (see Table 2). As expected, the effect of threshold criterion was significant for both upright and inverted faces: thresholds in the white noise condition were higher in the *t*_67_ group than the *t*_50_ group. After statistically controlling for the effect of criterion, the effect of orientation tuning was significant for upright but not inverted faces, and the interaction between criterion and tuning was not significant for either face orientation. These results indicate that tuning was correlated with identification thresholds for upright face but not inverted faces, and that that the correlation did not differ between the *t*_67_ and *t*_50_ groups. After combining the *t*_67_ and *t*_50_ groups, the Pearson correlation between log-transformed threshold and orientation tuning was −0.52 (*CI*_95_ = [−0.73, −0.21], *t*(30) = −3.33, *p* = 0.0023) for upright faces and −0.12 (*CI*_95_ = [−0.45, −0.24], *t*(30) = −0.65, *p* = 0.52) for inverted faces (Figure 6). Hence, greater orientation tuning was associated with lower identification thresholds for upright faces, but not inverted faces, in the white noise condition. Essentially the same results were obtained when the data were re-analyzed after removing the unusually low tuning score obtained with inverted faces from one subject in the *t*_67_ group (see Figure 4).

**Table 2:** **ANOVA tables for linear models predicting identification threshold in the white noise condition with upright faces (top) and inverted faces (bottom)**.

	df	Sum Sq	Mean Sq	F value	Pr(>F)
**UPRIGHT FACES**					
Criterion	1	0.91	0.91	7.80	0.009
Tuning	1	0.90	0.90	7.76	0.009
Criterion × tuning	1	0.01	0.01	0.09	0.762
Residuals	28	3.26	0.12		
**INVERTED FACES**					
Criterion	1	0.81	0.81	8.48	0.007
Tuning	1	0.02	0.02	0.26	0.616
Criterion × tuning	1	0.00	0.00	0.00	0.985
Residuals	28	2.67	0.10		

*The variable criterion refers to the criterion used to define threshold (i.e., the *t*_67_ and *t*_50_ groups); tuning refers to the orientation tuning of masking (see Figure 4). Type-I tests (i.e., sequential sums-of-squares) are shown: each row evaluates the change in the residual sum of squares that is produced by adding the predictor variable to a model that contains all of the predictor variables listed above it*.

**Figure 7:**
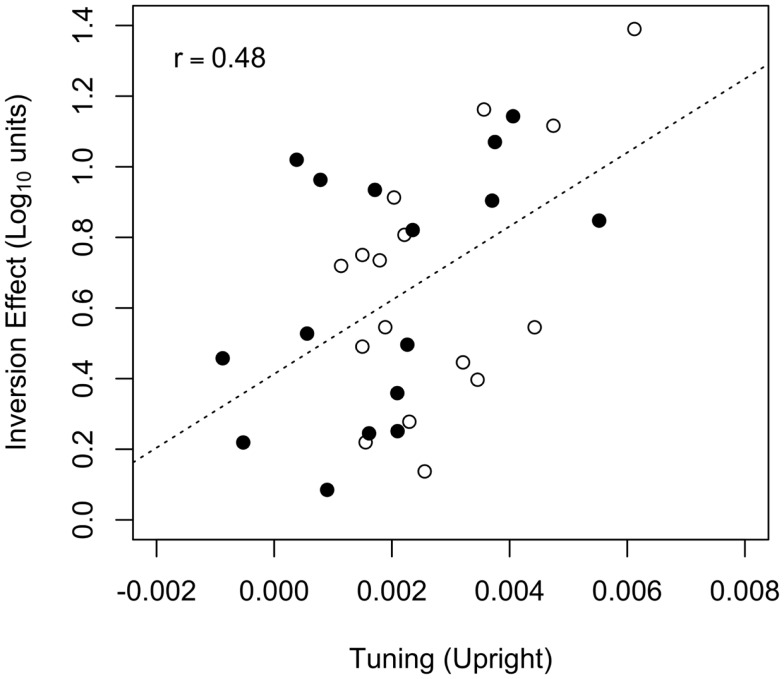
**The face inversion effect plotted against orientation tuning for upright faces**. Data from the *t*_67_ and *t*_50_ groups are represented by the filled and open symbols, respectively. The dotted line represents the best-fitting (least-squares) line fit to the data from both groups. The Pearson correlation between the face inversion effect and upright tuning (*r* = 0.48) was significant.

Given that orientation tuning predicted identification threshold for upright, but not inverted faces, it follows that orientation tuning may predict the size of the face inversion effect, defined here as the difference between the log-transformed contrast thresholds for upright and inverted faces. To assess the association between the face inversion effect and orientation tuning, we evaluated a linear model that used the face inversion effect as the dependent variable and threshold criterion, orientation tuning for upright faces, and the criterion × tuning interaction as predictor variables. The effect of orientation tuning was significant [*F*(1,28) = 9.14, *p* = 0.005], but the effects of threshold criterion [*F*(1,28) = 0.03, *p* = 0.86] and the criterion × tuning interaction [*F*(1,28) = 0.21, *p* = 0.65] were not. Because the effects of threshold criterion and the interaction were not significant, those two predictor variables were dropped from the model, and the best-fitting line relating upright face orientation tuning to the face inversion effect was computed: the face inversion effect was positively correlated with upright tuning (*r* = 0.48, CI_95_ = [0.16, 0.71], *t*(30) = 3.03, *p* = 0.005). Hence, greater orientation tuning for upright faces was associated with a larger face inversion effect (see Figure 7). A model using orientation tuning for inverted faces as a predictor variable fit the data poorly [${\text{R}}^{2}=0.015$, *F*(3,28) = 0.14, *p* = 0.933], and the face inversion effect was not associated with any of the predictor variables (*F* < 1 and *p* > 0.5 in all cases).

## 4 Discussion

In this experiment we found that human observers preferentially use horizontal information to identify upright faces more than inverted faces. This result is reflected in the masking functions of our human observers, as well as the significant linear regression between masking and noise orientation with upright faces. Moreover, our ideal observer analysis also obtained the strongest masking with horizontal noise, which suggests that more information relevant to face identification is carried in this band. Therefore, our findings suggest that human observers exploit diagnostic orientation information more efficiently when identifying upright faces. These results are consistent with previous demonstrations of a preference for horizontal information in upright but not inverted face discrimination (Goffaux and Dakin, 2010), and the presence of structured bands of horizontal information in face stimuli (Dakin and Watt, 2009). Our ideal observer analysis showed that information relevant to identification is carried at every orientation, with relatively more information available in the horizontal band. In fact, although our human observers demonstrated significant horizontal orientation tuning, particularly for upright faces, our absolute efficiency results indicate that, compared to other orientations, they were suboptimal in their use of the additional information carried in the horizontal band. However, this failure to take full advantage of the additional horizontal information is less pronounced with upright than inverted faces. Given these results, it follows that observers who best utilize horizontal information should also demonstrate the best overall face identification performance. Our results were consistent with this hypothesis: We found a significant negative correlation between orientation tuning and identification thresholds for upright faces but not inverted faces. We also found a significant correlation between orientation tuning for upright faces and the size of the face inversion effect.

Together, these results are consistent with recent demonstrations that the key difference between upright and inverted face processing is the manner in which observers encode horizontal structure (Goffaux and Dakin, 2010; Goffaux et al., 2011). However, we have demonstrated that face stimuli do indeed carry more diagnostic information in the horizontal band, and that differential sensitivity to this information explains much of the variance in upright face identification and the face inversion effect. Moreover, we find that observers are sensitive to information in the horizontal band when processing upright and inverted faces, but use this information less effectively with inverted faces. As such, the current results are consistent with the idea that the face inversion effect reflects quantitative differences in the efficiency with which observers extract diagnostic information from upright and inverted faces (Riesenhuber et al., 2004; Sekuler et al., 2004; Yovel and Kanwisher, 2004). Indeed, previous results using noise masking techniques have demonstrated only subtle differences in spatial sampling following inversion (Sekuler et al., 2004) or perceptual learning (Gold et al., 2004), coupled with changes in calculation efficiency (Gaspar et al., 2008a).

A great deal of information for face identification is clustered around the eye and eyebrow region (Gold et al., 1999, 2004; Sadr et al., 2003; Sekuler et al., 2004; Vinette et al., 2004; Gaspar et al., 2008b; Keil, 2009), and these regions are rich in horizontal structure (Dakin and Watt, 2009). Human observers likely learn to efficiently extract diagnostic information as they become experts with upright faces throughout their development (de Heering et al., 2012). Therefore, although horizontal information appears to be critical for upright face identification, in other tasks such as emotion discrimination or gender discrimination, different regions of the face or different orientations may be critical (Smith et al., 2005). It remains unclear whether orientation tuning is associated with behavioral performance in these tasks. Moreover, some aspects of face perception appear to differ across culture (Jack et al., 2009), age (Carey et al., 1980; Bruce et al., 2000; Mondloch et al., 2002; Boutet and Faubert, 2006; Habak et al., 2008; Rousselet et al., 2009; Obermeyer et al., 2012), specialized subject populations (Langdell, 1978; Archer et al., 1992; Duchaine and Nakayama, 2005), and contrast polarity (Vuong et al., 2005; Russell et al., 2006; Gaspar et al., 2008b). More work is needed to elucidate how orientation tuning may be associated with these phenomena. Moreover, it remains unclear whether orientation tuning can be modulated with perceptual learning. However, if orientation tuning is impaired in populations with impairments in face perception, and orientation tuning can be modulated with perceptual learning, then this line of research may prove fruitful in developing focused training programs to help ameliorate the deficits experienced by these individuals.

## Conflict of Interest Statement

The authors declare that the research was conducted in the absence of any commercial or financial relationships that could be construed as a potential conflict of interest.
